# Gelatinase-sensitive nanoparticles loaded with photosensitizer and STAT3 inhibitor for cancer photothermal therapy and immunotherapy

**DOI:** 10.1186/s12951-021-01125-7

**Published:** 2021-11-21

**Authors:** Lin-Lin Bu, Han-Qi Wang, Yuanwei Pan, Lei Chen, Hao Wu, Xianjia Wu, Chenchen Zhao, Lang Rao, Bing Liu, Zhi-Jun Sun

**Affiliations:** 1 The State Key Laboratory Breeding Base of Basic Science of Stomatology (Hubei-MOST) & Key Laboratory of Oral Biomedicine Ministry of Education, School & Hospital of Stomatology, Wuhan University, Wuhan, 430079 China; 2 Department of Oral & Maxillofacial-Head & Neck Oncology, School & Hospital of Stomatology, Wuhan University, Wuhan, 430079 China; 3 Institute of Biomedical Health Technology and Engineering, Shenzhen Bay Laboratory, Shenzhen, 518132 China

**Keywords:** Stimuli-responsive drug release, Indocyanine green, STAT3 inhibitor, Immunotherapy, Photothermal therapy

## Abstract

Matrix metalloproteinase (MMP) 2 and 9 are the family members of proteases normally up-regulated in tumor to enhance the invasion and metastatic of tumor cells, and are associated with poor outcome of head and neck squamous cell carcinomas (HNSCCs). In the present work, MMPs-degradable gelatin nanoparticles (GNPs) are simultaneously loaded with photosensitizer indocyanine green (ICG) along with signal transducer activator of transcription 3 (STAT3) inhibitor NSC74859 (NSC, N) for efficient photothermal therapy (PTT) and immunotherapy of HNSCCs. In the tumor tissue, Gel-N-ICG nanoparticle was degraded and encapsulated ICG and NSC were effectively released. Under near-infrared (NIR) irradiation, the released ICG nanoparticles enabled effective photothermal destruction of tumors, and the STAT3 inhibitor NSC elicited potent antitumor immunity for enhanced cancer therapy. Based on two HNSCC mouse models, we demonstrated that Gel-N-ICG significantly delayed tumor growth without any appreciable body weight loss. Taken together, the strategy reported here may contribute that the stimuli-responsive proteases triggered nanoplatform could reduce tumor size more effectively in complex tumor microenvironment (TME) through combination of PTT and immunotherapy.

**Supplementary Information:**

The online version contains supplementary material available at 10.1186/s12951-021-01125-7.

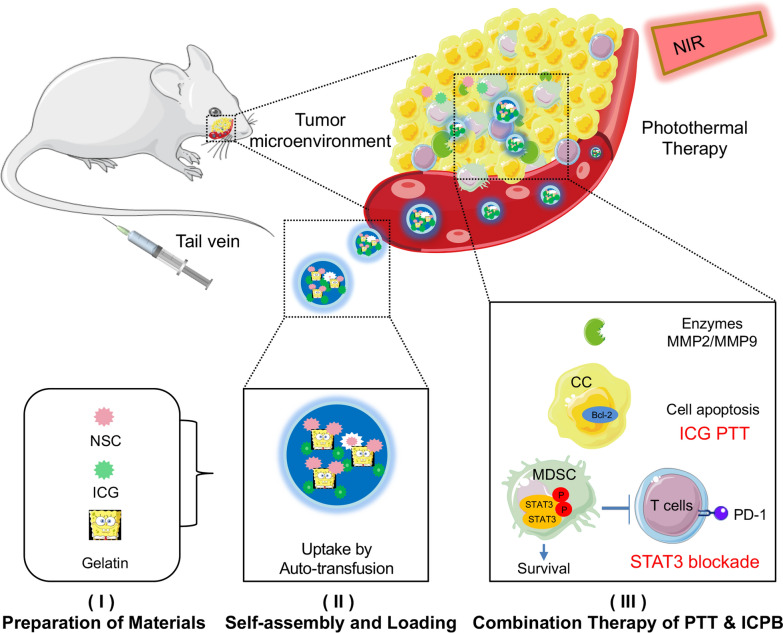

## Introduction

As the sixth most common cancer around the world, there are more than 930,000 new patients of head and neck squamous cell carcinomas (HNSCCs) in 2020, which account for about 90% of head and neck cancer patients [1, 2]. HNSCC is one of the most life-affecting malignant diseases, and except the deaths directly caused by HNSCC, the rate of suicide in HNSCC survivors (63.4 per 100,000 person-years) has been the second among all kinds of cancer [3]. Generally, current standard therapy of HNSCC still relies on surgery followed by conventional chemotherapy and radiotherapy [4, 5]. However, due to the dysfunction caused by surgery and the chemotherapy resistance, the outcome of HNSCC treatment strategies is usually accompanied by cancer recurrence, metastasis and poor prognosis, and the overall survival rate in past three decades has improved modestly [6, 7]. Nanotechnology demonstrated significant potential to reduce the side effects and increase drug targeting of immunotherapy [8]. So far, various nanomaterials have used in multiple fields including tumor treatment [9, 10]. Among them, nanomaterial-based drug carriers such as liposomes [11, 12], polymer [13, 14], inorganic and hybrid protein-inorganic nanoparticles (NPs) [15–17] have been developed as nanocarriers to deliver various drugs, e.g., chemical anti-tumor drugs [18, 19], enzymes [20], antibody [21, 22], siRNA [23, 24], noble metal [25, 26] in order to improve the therapeutic effect.

Gelatin, the earliest proteinaceous material in formation of NPs and the carriage of drug through intravenous infusion, exhibits excellent characteristics such as biocompatibility, biodegradability, non-immunogenicity and safety in medical using, can be degraded by gelatinase and ease of bridge [27]. Among the various gelatinases that overexpressed in tumor microenvironment, matrix metalloproteinase (MMP) has been demonstrated to have a promising effect in enriching gelatin NPs [28, 29]. Previous studies demonstrated that gelatin-based NP can be used as MMP responsive carrier for tumor cell imaging in cancer treatment [30, 31]. By loading with different drugs, gelatin NPs can play different role in inhibiting or diagnosing various cancers. For instance, Ruan S et al. designed a new way to improve targeting delivery efficiency and treatment outcome of glioma by loading gold NPs into gelatin NPs (GNPs) and decorating with doxorubicin (DOX) and Cy5.5 [32]. More recently, Chen et al. demonstrated the antitumor effect of GNPs encapsulating indocyanine green ICG and DOX in breast cancer [33].

As for drugs loaded in NPs, considering the immunosuppression and metastasis of cancer associated with the overexpression of programmed cell death ligand 1 (PD-L1) induced by constitutive activation of STAT3, NSC74859 (NSC, N), a signal transducer activator of transcription 3 (STAT3) inhibitor targeting Src Homology 2 (SH2) domain, was used to delay the growth of HNSCC in our previous works [34]. Moreover, indocyanine green (ICG), approved by FDA in clinical use, is a near-infrared dye and widely used in fluorescence imaging (FI) and photothermal therapy (PTT) [35, 36]. As the novel way in cancer treatment, principle of PTT is converting light to heat by exposing photosensitive nanoparticles to laser which can induce the tumor cell elimination and enhance the effect of immune agents via the transformation of the immunosuppressive “cold” tumor to the immunosensitive “hot” tumor [37, 38].

**Scheme 1:**
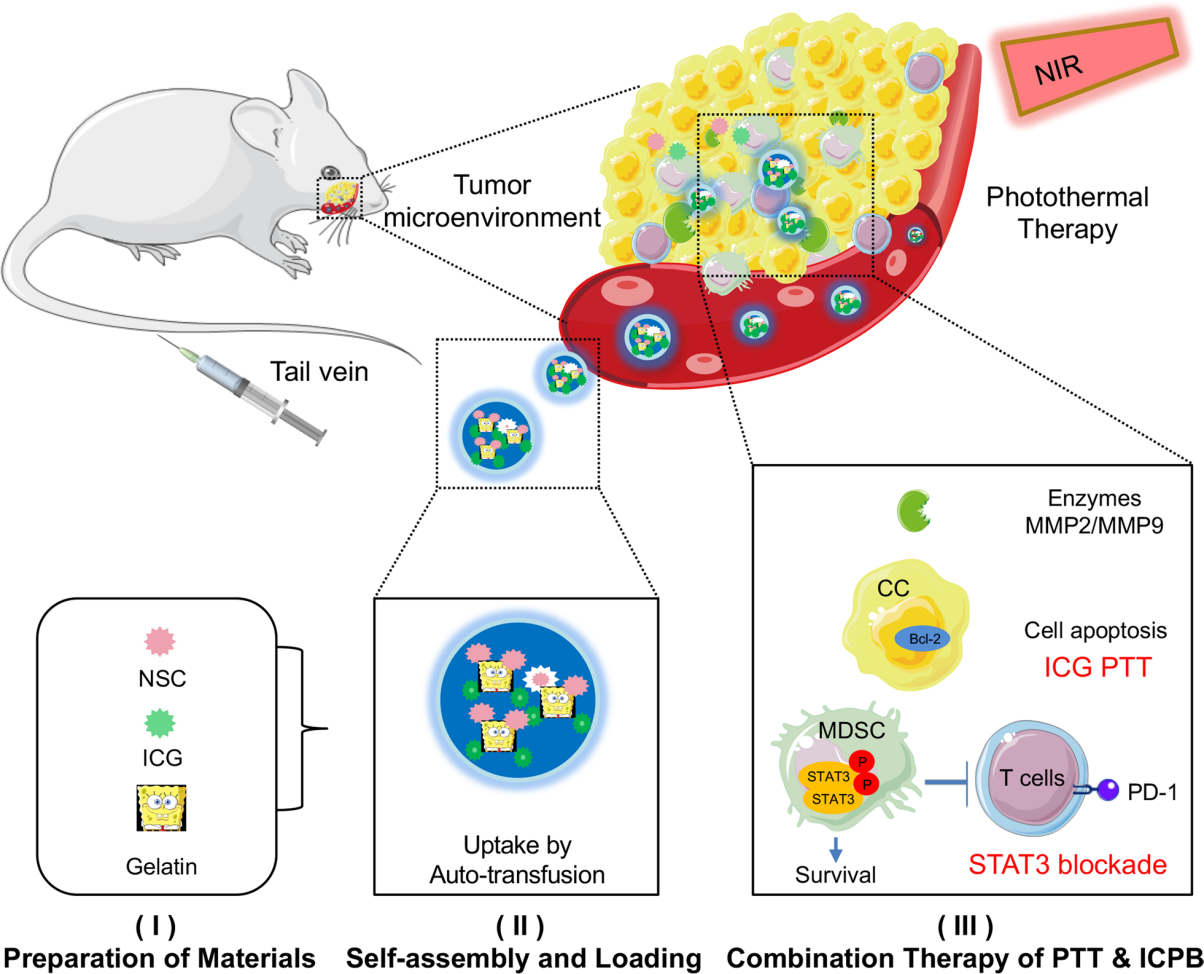
**I**, **II** Preparation of ICG and NSC encapsulated supramolecular gelatin nanoparticles (Gel-N-ICG NPs) and uptake through i.v. injection; **III** schematic representation of PTT and multi-functional immunotherapy by Gel-N-ICG-NPs in the treatment of HNSCC

Herein, we developed a self-assemble gelatinase sensitive nanoparticle loaded with ICG and NSC (Gel-N-ICG) to achieve targeted drug delivery and HNSCC inhibition through both PTT and mufti-functional immunotherapy (Scheme 1). The supramolecular gelatin NPs (SGNPs) were prepared via a desolvation technique, ICG and STAT3 inhibitor NSC were co-immobilized into GNPs. Subsequently, the resulting Gel-N-ICG NPs accumulated in tumor site via the enhanced permeability and retention (EPR) effect after intravenous (i.v.) injecting into the donor mice [39]. When NPs arrived at targeting region, the drug delivery system were degraded by MMP with the release loaded drugs. The released ICG played the role in PTT as the photothermal adjuvant after the absorption in wavelength of 808 nm. Meanwhile, released NSC played a role in immunotherapy through blocking the immune checkpoint protein and inducing the immune respond.

## Results and discussion

### Preparation and characterization of Gel-N-ICG nanoparticles

In this study, we synthesized the Gel-N-ICG NPs via desolvation technique according to previously studies, which has been demonstrated to be efficient in small nanoparticles formation [30, 40]. The drug loading was achieved by embedded the ICG and NSC into the hydrogel-like interior of SGNPs. The Gel-N-ICG NPs were prepared through self-assembly process on a microfluidic platform, the physiochemical parameters of Gel-N-ICG NPs including size, surface charge, stability can be controlled precisely and reproducibly by changing flow rates of fluids containing gelatin, ICG and NSC.

**Fig. 1:**
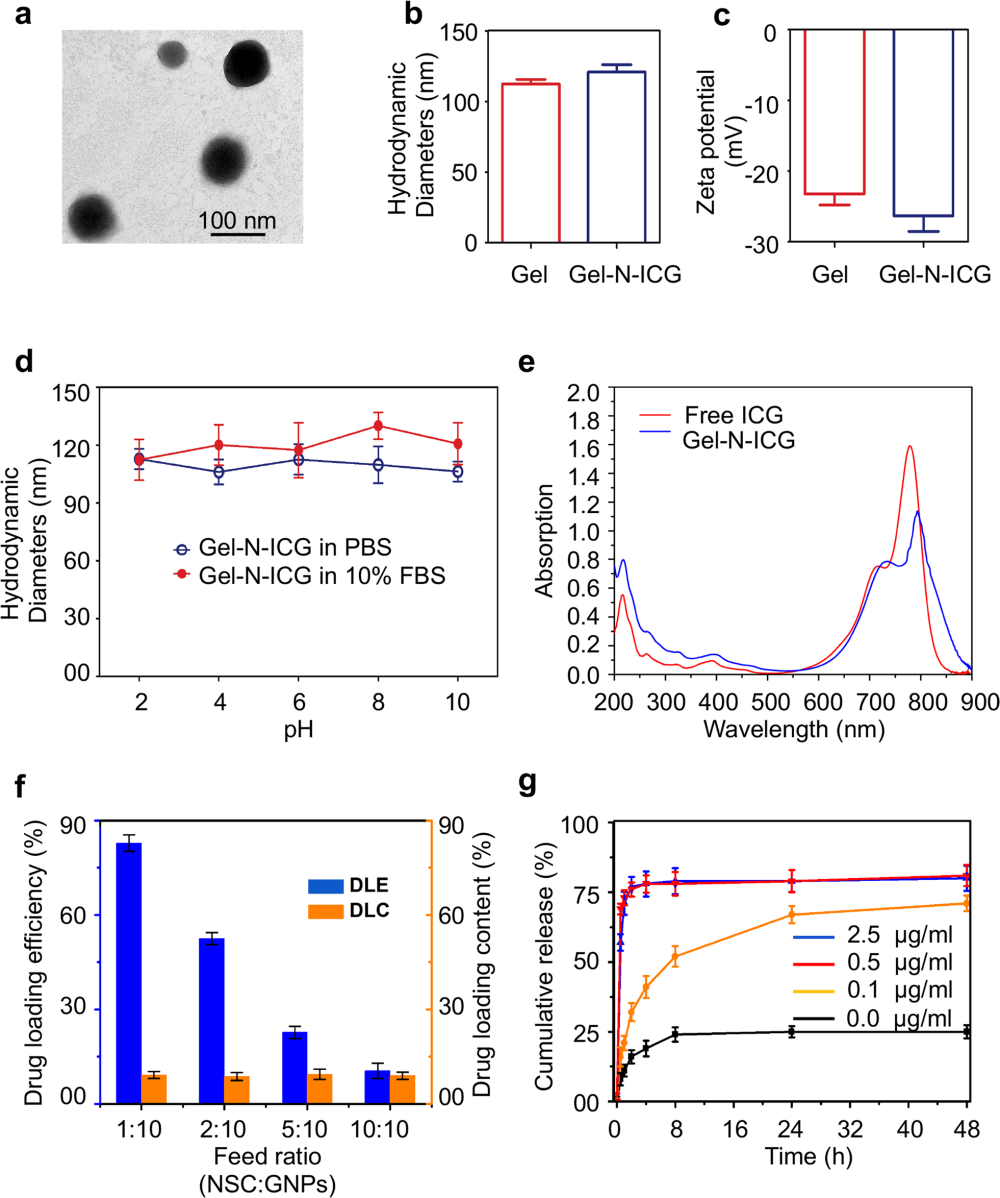
Physicochemical characterization of Gel-N-ICG-NPs, loading efficiency, loading content, and release curve of NSC. **a** Representative TEM images of Gel-N-ICG-NPs. **b** Mean diameter size of bare Gel-NPs and Gel-N-ICG-NPs (n = 3, mean ± SD). **c** Zeta potential of Gel-NPs and Gel-N-ICG-NPs (n = 3, mean ± SD). **d** Stability studies of Gel-N-ICG-NPs under different pH values from 2 to 10 in PBS buffer and DMEM containing 10% FBS, respectively. **e** UV–vis–NIR spectra of Gel-N-ICG and free ICG which indicated the drug loading. **f** DLE and DLC of NSC in Gel-N-ICG-NPs. **g** Time course of drug release from Gel-N-ICG-NPs in different concentrations of gelatinase

Transmission electron microscopy (TEM) was used to visualize the morphology of Gel-N-ICG NPs. As TEM image revealed, Gel-N-ICG NPs was about 100 nm with a spherical shape (Fig. 1a). Compared to SGNPs, the hydrodynamic diameters of Gel-N-ICG NPs measured by dynamic light scattering (DLS) were demonstrated to an increase due to the loading of NSC and ICG inside the SGNPs (Fig. 1b). Change in zeta potentials of SGNPs after loading with NSC and ICG was also presented in Fig. 1c. Moreover, in order to evaluate the stability of Gel-N-ICG NPs, the NPs were suspended in PBS buffer and DMEM containing 10% FBS with different pH values from 2 to 10 and measured the size by DLS. As shown in Fig. 1d, DLS of Gel-N-ICG NPs in two different mediums with different pH had no significant difference which demonstrated the stability of these NPs. To confirm the successful loading of ICG into Gel and explore the ideal wavelength for PTT, Gel-N-ICG-NPs and free ICG were exposed into laser irradiation with various wavelength (Fig. 1e) and the results demonstrated that Gel-N-ICG-NPs and free ICG all exhibited a characteristic absorption peak at about 808 nm.

### In vitro and in vivo toxicity studies

We then investigated the cytotoxicity of Gel and ICG in vitro. The cell viability of HNSCC cell line CAL27 and normal epithelial cell line HIOEC were monitored after treating with different concentrations of Gel or ICG. And the results did not present a significant change which demonstrated the safety of Gel and ICG (Additional file 1: Fig. S1). Moreover, the nanoparticle systems are of site-specific drug accumulation in tumors and have been reported as promising ways to overcome the potential in vivo toxicity of chemotherapy [41]. To test the potential toxicity of ICG and Gel-NPs in vivo, 15 ICR mice (*n *= 5) received an i.v. injection of 200 µL PBS, or PBS containing ICG, Gel-NPs at the concentration of 5 mg mL^−1^. After 30-day treatment, no death or significant difference in body weight was observed among ICG and Gel-NPs groups compared to the control group (Additional file 1: Fig. S2), which demonstrated that treatment with ICG and Gel-NPs have no obviously overall side effects. Moreover, blood and major organs of all ICR mice including heart, liver, spleen, lung and kidney were collected on the 30th day for the blood biochemistry test and H&E staining. Their blood parameters and H&E-stained slice images showed no significant differences between treated groups and control group (Additional file 1: Table S1, Fig. S3), which further demonstrated the safety of ICG and Gel-NPs application in vivo.

### Drug loading assay and enzyme-triggered release of Gel-N-ICG NPs

To optimize the reaction conditions, the effect of drug loading and release in different weight ratio of NSC to SGNPs and concentration of gelatinase were evaluated. To measure parameters of drug loading, SGNPs (10 mg mL^−1^) with weight ratio of NSC to SGNPs of 1:10, 2:10, 5:10, and 10:10, respectively, were quantified after swelling and loading of NSC into gelatin NPs for 24 h. As presented in Fig. 1f, a higher drug loading efficiency (DLE) was achieved when the weight ratio of NSC to SGNPs was 1:10 while drug loading content (DLC) of NSC in Gel-N-ICG NPs had no significant difference between treated groups. And 1:10 was regarded as the optimized ratio of NSC to SGNPs with which the maximum absorption of NSC and SGNPs can be achieved. In the gelatinase solution, gelatin from encapsulated Gel-N-ICG NPs was degraded and allowed the release of NSC. As presented in Fig. 1g, in pH = 7.4 PBS buffer solutions, different concentrations of gelatinase effected the cumulative release of drug from Gel-N-ICG-NPs. It was demonstrated the increase of total cumulative release with the increasing concentration of gelatinase (at the range of 0.0–0.5 µg/mL). And after 4 h, the drug in 0.5 and 2.5 µg/mL gelatinase achieved a maximum release of more than 75%.

### In vitro antitumor effect of Gel-N-ICG NPs

**Fig. 2:**
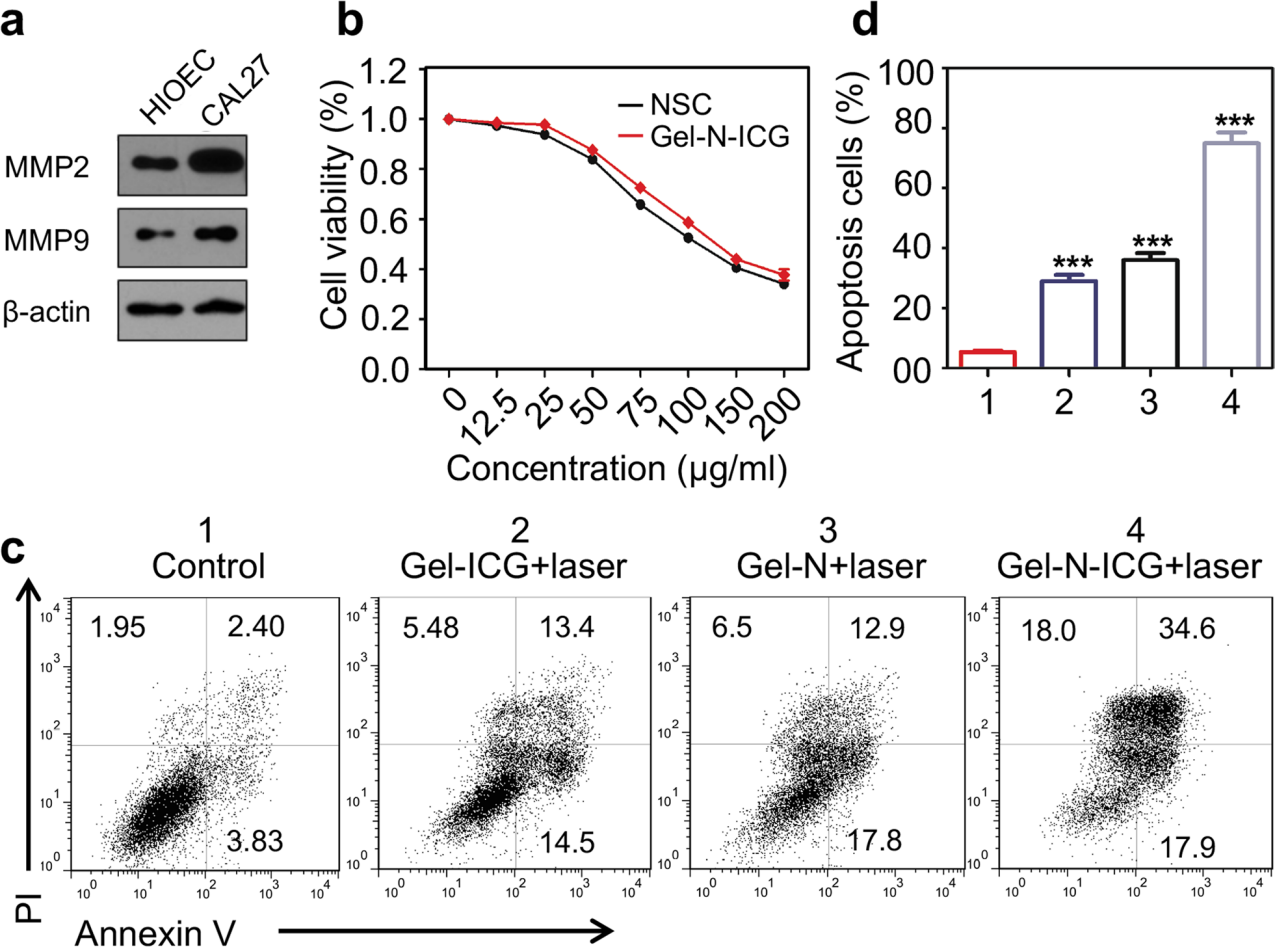
Cell survivals of CAL27 cells after chemo-photothermal treatment. **a** Western blot qualitative in CAL27 and HIOEC. **b** Cell viability in different concentration of NSC and Gel-N-ICG NPs. **c** Flow cytometry analysis of CAL27 cells after different treatments. **d** Quantitative apoptosis cells (%) in different treatment groups for 24 h. *, ** and *** indicate *P* < 0.05, *P* < 0.01 and *P* < 0.001, respectively, as compared with the PBS group

Moving onward, we performed western blot and conducted a meta-analysis in TCGA data base to assess the protein expression of MMP9 and MMP2. The results displayed the overexpression of MMP9 and MMP2 in HNSCC cell line CAL27 compared to normal human epithelial cell line HIOEC (Fig. 2a, Additional file 1: Fig. S4), which will facilitate the degradation of gelatin NPs. Cell viability of CAL27 exhibited a remarkable regression as the increasing concentration of NSC which showed the anti-tumor efficacy of NSC. And there was no significant difference in cell viability between the treatment with NSC and Gel-N-ICG NPs without laser irradiation (Fig. 2b). To demonstrate the anti-tumor effect of immuno-photothermal therapy which combined Gel-N-ICG NPs with laser irradiation in HNSCC cells, CAL27 was treated with various solutions. Standard CCK-8 assay and Annexin V/PI staining were performed to show the apoptosis of CAL27 without any drug and after treatment with various solutions including Gel-ICG, Gel-N and Gel-N-ICG with laser irradiation for 24 h (Fig. 2c, d). As presented in flow cytometry analysis, cytotoxicity was mostly observed in Gel-N-ICG with laser irradiation treated group compared to other treated groups. And the apoptosis of CAL27 in vitro was significantly higher in treated groups compared to control group (*P* < 0.001).

### In vivo antitumor efficacy of Gel-N-ICG NPs in nude mice HNSCC model

**Fig. 3:**
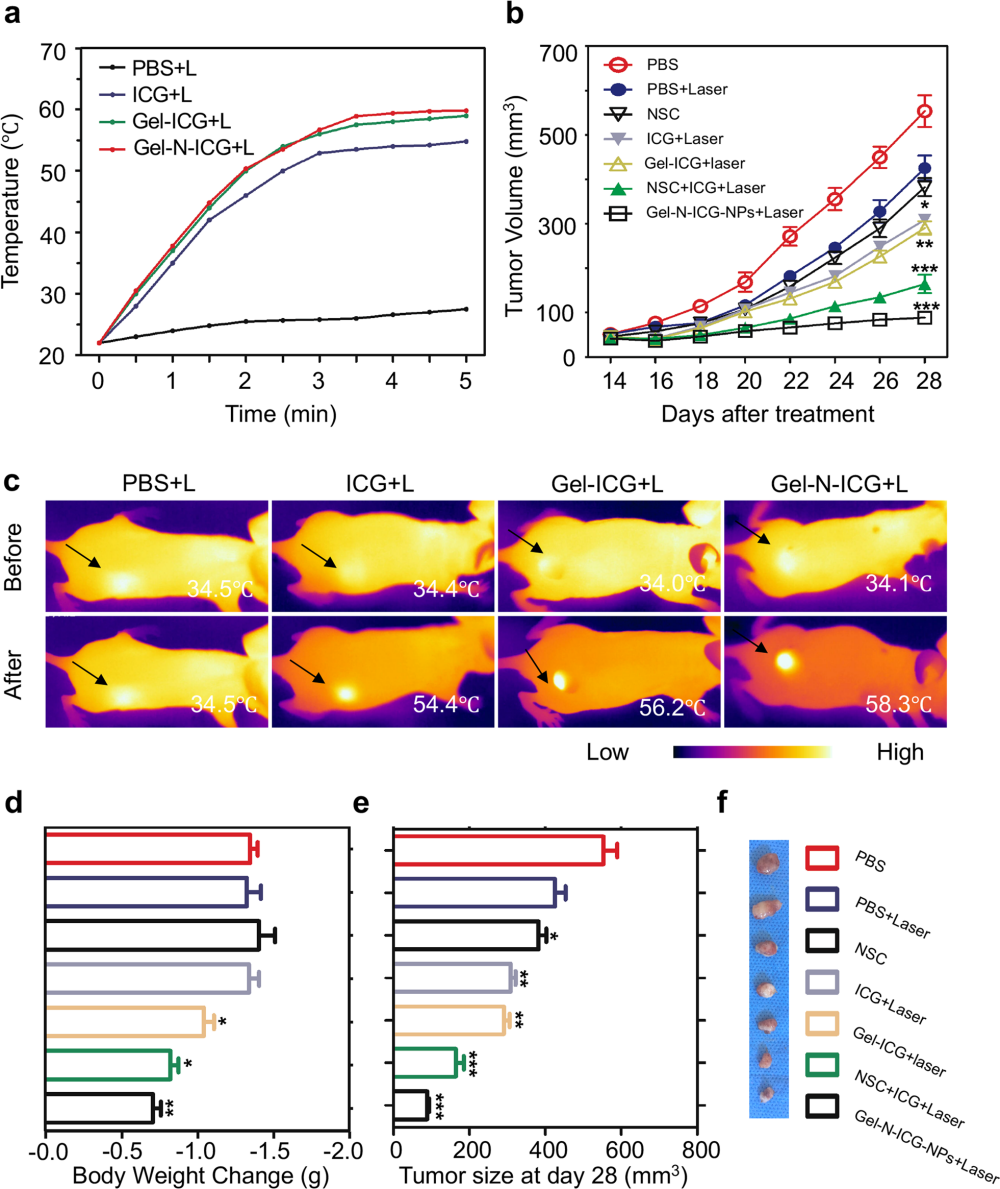
Tumor therapy with Gel-N-ICG in xenograft mice model. **a** Maximum temperature profiles of the tumor site of nude mice after injection with different solutions with or without the 808 nm laser irradiation. **b** Tumor volume curves over 28 days after different treatment. **c** Representative in vivo IR thermal images of xenograft mice before and after injection with different solutions with a 5-min exposure to the laser irradiation. **d** Tumor bearing mice weight change, **e** tumor size at day 28 and **f** representative ex vivo tumor images from mice in different treatment groups. All data points represent as mean ± SD (n = 5). NS, *, **, and *** indicate no statistical difference, *P* < 0.05, *P* < 0.01, and *P* < 0.001, respectively, as compared with the PBS group

To evaluate the anti-tumor efficacy of Gel-N-ICG NPs in vivo, BALB/c nude mice bearing CAL27 tumor xenografts were treated with various solutions including PBS, ICG, Gel-ICG, and Gel-N-ICG with the exposure to an 808 nm laser. The temperature of the tumor site in treated group gradually increased during the 5 min presence of laser irradiation which was attributed to the absorption of ICG at 808 nm laser, while no increase of temperature was observed in control group nearly (Fig. 3a). As images taken by infrared (IR) thermographic camera showed (Fig. 3c), the temperature increased to 54.4, 56.2, 58.3 °C, respectively, within 5 min laser irradiation in different treated groups. Furthermore, changes in tumor volume of nude mice models were regarded as the best direct index in evaluating the anti-tumor efficacy. As presented in Fig. 3b, tumor volume of each mouse was measured every other day from 14 day after various treatments. And on 28th day after treatment, all the tumor bearing nude mice were euthanized for the further experiments. Tumor volume in Gel-N-ICG with laser group achieved a remarkable control at 28th day after treatment, while tumor volumes in PBS, Gel-ICG, Gel-N, NSC, NSC-ICG with or without laser control groups presented a rapidly growth, which suggested that Gel-N-ICG NPs with laser can obtain the significant chemical and photothermal elimination of tumor compared to other groups (Fig. 3e, f). The body weights of mice at day 28 in Gel-N-ICG with laser group shown the least impacted compared to control group, which demonstrated the safety and anti-tumor effect, while in PBS with laser, ICG with laser and NSC groups, the body weights of mice showed a significantly regression (Fig. 3d).

### In vivo anti-tumor effect of Gel-N-ICG NPs in *Tgfbr1/Pten* 2cKO mice HNSCC model

**Fig. 4:**
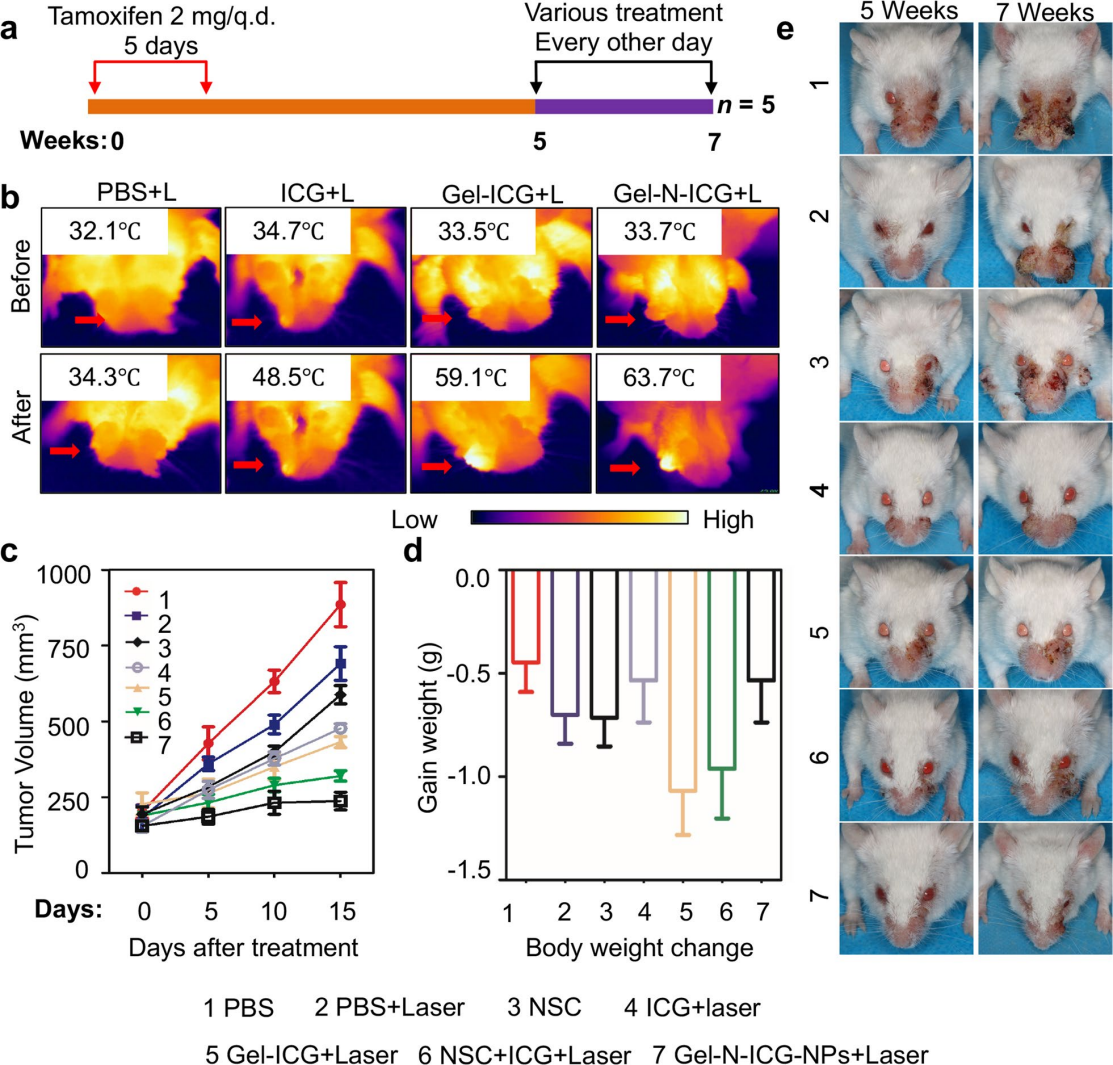
In vivo tumor therapy with Gel-N-ICG in *Tgfbr1/Pten* 2cKO mice HNSCC. **a** Schematic of the *Tgfbr1/Pten* 2cKO mouse model treatments strategy. Tamoxifen was applicated into oral consecutively for 5 days. 5 weeks after application, HNSCC induced mice consecutively treated with various strategies every other day for 15 days. Data present as mean ± SD, n = 5, respectively. **b** Representative in vivo IR thermal images of 2cKO mice before and after injection with different solutions with a 5-minute exposure to the laser irradiation. **c** Tumor volume curves over 15 days in different treatment groups. **d** Body weight change of 2cKO mice in different groups on 15th day after treatment. **e** Representative ex vivo tumor photo of 2cKO mice before and after treatment. All data points represent as mean ± SD (n = 5)

Bian et al. demonstrated that there were similar pathology and multiple molecular alterations of head and neck carcinogenesis between human and *Tgfbr1/Pten* double conditional knockout (2cKO) mice, so *Tgfbr1/Pten* 2cKO mouse was regarded as a suitable model in HNSCC tumorigenesis [42]. Here, we conducted the *Tgfbr1/Pten* 2cKO mice model to further evaluate the anti-tumor effect and immune response of Gel-N-ICG NPs in vivo. As HE stain confirmed (Additional file 1: Fig. S5a), tumor tissue of *Tgfbr1/Pten* 2cKO mice was similar to HNSCC tissue derived from human [42]. The expression level of gelatin enzyme including MMP2 was detected by immunohistochemically staining, as it presented in Additional file 1: Fig. S5b, MMP2 was overexpressed in *Tgfbr1/Pten* 2cKO mice HNSCC compared to normal tissue. To evaluate the anti-tumor effect, *Tgfbr1/Pten* 2cKO mice received oral application of 2 mg tamoxifen for consequent 5 days. On 30th day after induction, these *Tgfbr1/Pten* 2cKO mice with squamous cell carcinoma growing in their oral cavity were treated with i.v. injection of various drugs with or without laser every other day for 15 days (Fig. 4a). As IR thermal images presented in Fig. 4b, the temperature of tumor site of *Tgfbr1/Pten* 2cKO mice raised from 33.7 to 63.7 °C in the group of Gel-N-ICG with 808 nm laser irradiation for 5 min, which was higher than the 2.2 °C-increase in the group of PBS with laser irradiation. As presented in Fig. 4c, tumor volume in group 7 (Gel-N-ICG+ Laser) exhibited a significant reduction 15 days after application compared to tumor volume in group 1 (PBS) (*P* < 0.001). Moreover, there was no significant change in body weight of HNSCC mouse model between PBS control group and Gel-N-ICG group, which indicated that no systemic side effect was produced during the treatment combing PTT with immunotherapy (Fig. 4d). The representative tumor photo ex vivo before and after treatment (Fig. 4e) directly demonstrated that Gel-N-ICG NPs exposed into laser caused a remarkable tumor damage and inhibit the growth of tumor during the 2-week treatment.

### Immune response of Gel-N-ICG NPs in *Tgfbr1/Pten* 2cKO mice HNSCC model

**Fig. 5:**
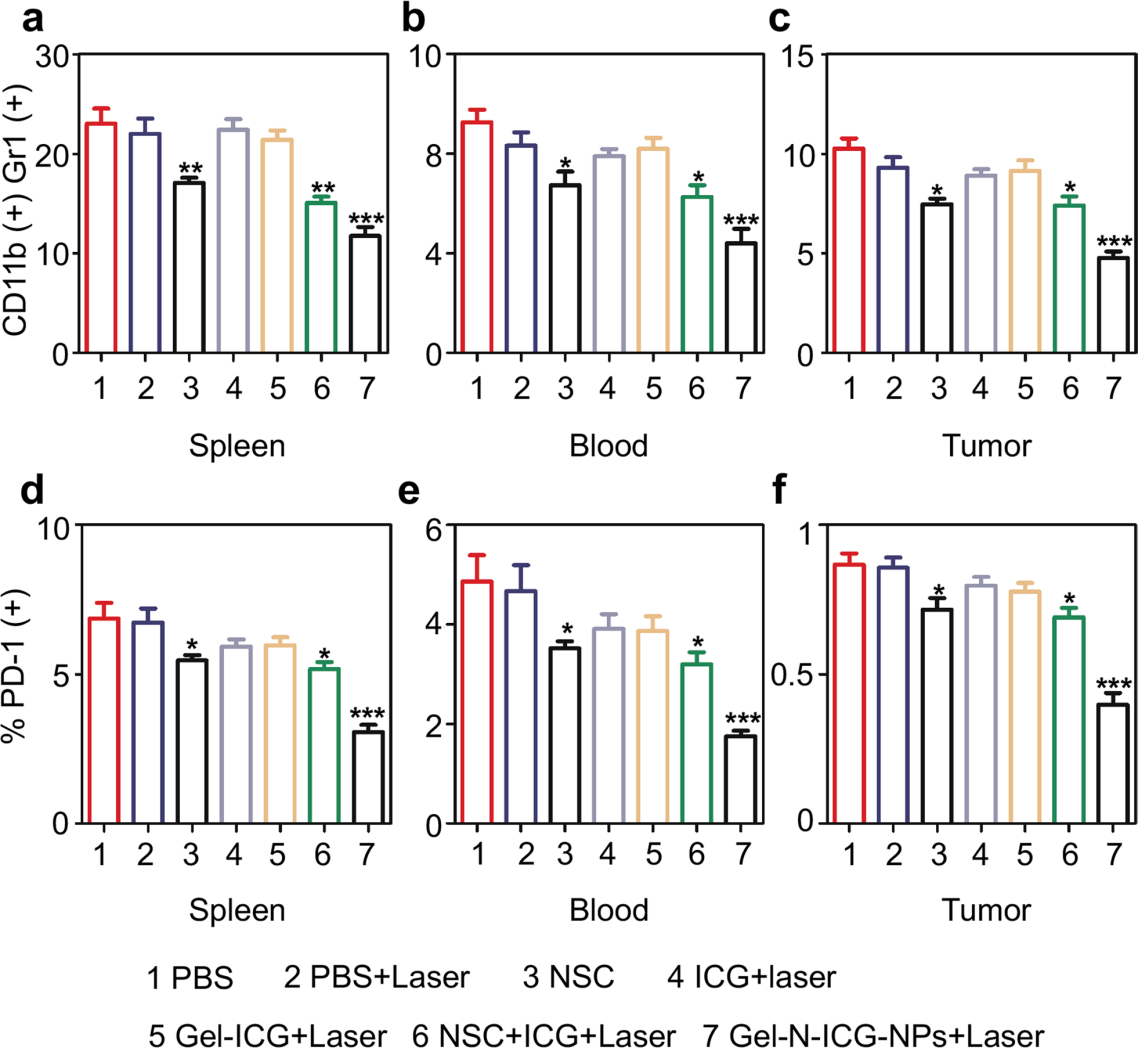
The population of MDSCs and PD-1 in *Tgfbr1/Pten* 2cKO mice. **a**–**c** Quantification of the percent of CD11b^+^ Gr1^+^ MDSCs in spleen, blood, tumor from mouse model in different treatment groups. **d**–**f** Quantification of the percent of PD-1^+^ cells in spleen, blood, tumor from mouse model in different treatment groups. (Data presented as mean ± SD, n = 5 mice respectively. NS, *, **, and *** indicate no statistical difference, *P *< 0.05, *P *< 0.01, *P *< 0.001, as compared with the PBS group)

To explore the immune response of Gel-N-ICG NPs, *Tgfbr1/Pten* 2cKO mice were euthanized on 15th day after various treatments, and the tumor tissues, blood and spleen of mouse were harvested and analyzed through flow cytometry. CD11b^+^ Gr1^+^ myeloid-derived suppressor cells (MDSCs) enhance the ability of tumor in immune evasion, invasion to the vasculature system and angiogenesis, which were associated with growth and metastasis of solid tumors [43]. As representative flow cytometry profiles shown in Fig. 5a–c, in Gel-N-ICG with laser group, the number of CD11b^+^ Gr1^+^ MDSCs in spleen, blood and tumor tissue were significantly decreased compared to PBS control group, which demonstrated the enhancement of anti-tumor immune response after treatment with Gel-N-ICG NPs exposed to laser irradiation. Moreover, the activation of immune response was further confirmed by flow cytometry (Fig. 5d–f), the population of programmed cell death protein 1 (PD-1), the inhibitory receptor expressed on all T cells and limited protective immunity of T cells, presented a remarkable decrease in Gel-N-ICG NPs+ Laser group as well [44]. According to these results, Gel-N-ICG NPs with laser irradiation was demonstrated to have the ability to inhibit the immunosuppression of tumor microenvironment (TME), which can enhance the anti-tumor efficacy.

## Conclusions

In summary, we have successfully created a gelatinase sensitive nanoparticle encapsulated ICG and STAT3 inhibitor (Gel-N-ICG) for efficient immunotherapy and PTT. Size-controllable SGNPs were easy to obtain. The Gel-N-ICG NPs performed good blood biochemistry, hematology tests, and histology analysis results which suggested no obvious toxicity occurred in the gelatin-based NPs in vitro and in vivo*.*

Meanwhile, the gelatin was degraded by MMP2/9, the gelatinase overexpressed in tumor microenvironment, and the encapsulated NSC and ICG were released in tumor site subsequently. Moreover, compared to the HNSCC xenograft nude mice model, the immune response, temperature of tumor surface after PTT and anti-tumor efficacy of Gel-N-ICG NPs were enhanced in *Tgfbr1/Pten* 2cKO head and neck carcinoma mice model which was of a closer TME to human HNSCC.

This approach has demonstrated an innovative anti-tumor delivery system for treating HNSCC. And this study may be proved helpful in reducing the solid tumor growth and increasing the efficacy of PTT and antitumor strategy.

## Methods

### Cells, animal models and human HNSCC tissues

CAL27 human squamous carcinoma cells were purchased and cultured as we reported before [34]. HIOEC (normal human epithelial cells) were used as a normal control. Two mouse models including ICR mice, BALB/c nude mice were purchased as previously reported and according to this previously study, we used CAL27 tumor cells to obtain the HNSCC xenografts [12]. And when the tumor size was at about 50 mm^3^, these xenograft mice were used for further studies. The inducible tissue-specific immunocompetent *Tgfbr1/Pten* 2cKO mice were obtained and maintained as previous described [42]. All the animal studies were approved by the Experimental Animal Ethics Committee of the School and Hospital of Stomatology at Wuhan University. Human HNSCC tissue with clinicopathological data and follow up was used for immunohistochemistry staining as previous study [45]. This study was approved by the Medical Ethics Committee of School and Hospital of Stomatology of Wuhan University and tumor tissues were derived from patients diagnosed with HNSCC.

### Materials and reagents

Gelatin type B (225 bloom) from bovine skin, selenium (99%, powder), sulfur (99.9%, powder), glutaraldehyde solution (Grade I, 50%) were obtained from Sigma Aldrich. Trioctylphosphine (TOP, 90%) was obtained from Acros Organics. SU-8 2050 photoresist was purchased from MicroChem, USA. RTV615 Silicone Potting Compound was obtained from Momentive Performance Materials (Waterford, NY, USA). Recombinant human tissue inhibitor of metalloproteinases 2 (TIMP2) was obtained from Sino Biological (Beijing, China).

### Synthesis of Gel-N-ICG NPs, drug loading, release and nanoparticle characterization

The SGNPs were prepared *via* desolvation method according to the previous studies [30]. The method to achieve the loading of NSC inside the gelatin NPs and the measure of the morphology, structure, ultraviolet–visible–NIR absorbance spectra, dynamic diameters of nanoparticles, the encapsulation efficiency of NSC and ICG were referred to the previous literature [40]. Firstly, the NSC of which the concertation was 10 mg mL^−1^ with different weight ratio to SGNPs of 1:10, 2:10, 5:10, and 10:10, respectively, was added into synthesized SGNPs. Then, after a 24-h swell and loading at RT, the unloaded NPs were removed by dialysis (Spectra/Por 4, MWCO 12,000 to 14,000) against DI water overnight to purify NPs. After the dialysis of NPs, the concentration of free NSC and the DLE and DLC of NSC was measured via the mothed was described in previous literature, and to measure the release of NSC from Gel-N-ICG NPs in PBS buffer solutions with different concentration of gelatinase (pH = 7.4), the HPLC was used to determine the Gel-N-ICG NPs release profiles as previous literature [40].

### Cell proliferation assay and Annexin V/PI staining

To evaluate the cell proliferation, CCK8 assay and Annexin V/PI staining was conducted following the manufacture’s instruction, and proliferation of cells were counted by flow cytometry.

### Dynamic light scattering (DLS)

We used a Zetasizer Nano instrument (Zetasizer Nano ZS, Malvern Instruments Ltd., UK) with a 10-mW He-Ne laser and a thermoelectric temperature controller at the temperature of 25 °C and detection angle of 90° to measure the hydrodynamic particle size. The data were processed subsequently by the Dispersion Technology Software (Malvern Instruments Ltd. UK).

### In vivo toxicity evaluation

The potential in vivo toxicity of Gel and ICG was evaluated through i.v. injection of 200 µL PBS, PBS containing ICG, PBS containing Gel with the concentration of 5 mg mL^−1^ into 15 ICR mice (*n *= 5), respectively. And every 3 days, we measured the body weights of treated mice. After the injection of solutions for 24 days, we euthanized the treated mice and collected their blood and major organs to measure the blood panel data and observed the organs sections stained with HE. The healthy mice were used as the control group.

### In vivo PTT evaluation

35 mice bearing SCC (n = 5) received i.v. injection of 100 µL PBS or PBS with laser (1 W cm^−2^, 5 min), NSC, ICG with laser, Gel-ICG with laser, NCS with ICG with laser and Gel-N-ICG NPs plus laser at the concentration of 5 mg NSC kg^−1^. During the PTT, tumor sites were measured with an IR thermographic camera (FORTRIC225, Shanghai Thermal Image Electromechanical Technology Co. Ltd, China). After the PTT, tumor volumes of mice were measured every other day. On the 28th day after treatment, the changes of body weight of mice were measured and all mice were euthanized to collect the tumors. Then, the collected tumors were sectioned into 4 μm and observed after staining using Ki-67.

*Tgfbr1/Pten* 2cKO mice were treated with a 5-day oral application of 2 mg tamoxifen every day. On the 30th day after induction, SCCs were inducted in the mice oral cavity. To further compare the tumor of 2cKO mice with the human HNSCC, representative mice were euthanized to collect the tumor. Subsequently, total 35 2cKO mice (n = 5) received the i.v. injection of 100 µL PBS or PBS with laser (1 W cm^−2^, 5 min), NSC, ICG with laser, Gel-ICG with laser, NCS with ICG with laser and Gel-N-ICG NPs plus laser at the concentration of 5 mg NSC kg^−1^. During the PTT, IR thermographic camera was used to record the temperatures of the tumor. After the PTT, the tumor volumes of treated mice were measured every 5 days. At the 15th day after treatment, the changes of body weight were measured and all mice were euthanized to collect the tumor for the further experiments.

### Flow cytometry analysis

Single cell suspensions from tumor site, spleens, and blood of *Tgfbr1/Pten* 2cKO mice with a variety of treatments were prepared. Tamoxifen inducted wide-type mice were set for flow cytometry analysis. And FITC-conjugated anti-mouse CD11b antibody, PE-conjugated anti-mouse PD-1 and Gr-1 antibody were used to label these cells. FACS of these cells was conducted via flow cytometer.

### Statistical analyses

Graph Pad Prism for Windows was used as introduced before for statistical analyses [12]. The difference among groups were detected by one-way ANOVA followed by the post-Tukey multiple comparison tests. Dates were represented as the mean ± SD. Differences (*P* < 0.05) were of statistical significance.

## Supplementary Information

**Additional file 1: Table S1.** Complete blood panel analysis of mice in the control and ICG, Gel-NPs.Standard deviations are based on 4 mice per group. WBC: white blood cell, RBC: red blood cell, HGB: hemoglobin, HCT: hematocrit, MCV: mean corpuscular volume, MCH: mean corpuscular hemoglobin, MCHC: mean corpuscular hemoglobin concentration, PLT: platelets, RDW: red blood cell distribution width. **Figure S1.** In vitrocytotoxicity of Gel and ICG against HNSCC cell line CAL27 and normal epithelialcell line HIOEC. (a, c) Cell viability of CAL27 cells with treatment with aseries of concentrations of Gel. (b, d) Cell viability of CAL27 cells withtreatment with a series of concentrations of ICG. **Figure S2.** Body weight change curves over a span of 30 d after the i.v.injection of PBS, ICG and Gel-NPs. **FigureS3.** Representative HE stained major organ slice images of mice 30 d after repeating i.v.injection of PBS, ICG and Gel-NPs; scalebars, 50 µm. **Figure S4.** Expression of Enzyme in Human HNSCC. (a, b) Meta-analysis of recent gene expression profiling for MMP2 and MMP9 by TCGA database (n = 566, HNSCC = 522, Normal = 44). P < 0.001.(c) Over-expression of MMP2 in head and neck squamouscell carcinoma as compared with normal oral mucosa (n = 3); scale bars, 50 µm. **Figure S5.** Gelatin enzyme expression in *Tgfbr1/Pten* 2cKO mice. (a) Representative HE staining of *Tgfbr1/Pten* 2cKO head and neck squamous cell carcinoma tissue (n = 3); scale bar, 50 µm. (b) Immunohistochemically staining indicate increase MMP2 expression in *Tgfbr1/Pten *2cKO mice HNSCC (n = 3); scalebars, 50 µm.

## Data Availability

All data generated or analyzed during this study are included in this published article and the additional information.

## Abbreviations

MMP: Matrix metalloproteinase
HNSCCs: Head and neck squamous cell carcinomas
GNPs: Gelatin nanoparticles
ICG: Indocyanine green
NSC: NSC74859
PTT: Photothermal therapy
NIR: Near-infrared
DOX: Doxorubicin
FI: Fluorescence imaging
STAT3: Signal transducer activator of transcription 3
SH2: Src homology 2
EPR: Enhanced permeability and retention
TEM: Transmission electron microscopy
DLS: Dynamic light scattering
DLE: Drug loading efficiency
DLC: Drug loading content
IR: Infrared
TUNEL: Terminal deoxynucleotidyl transferase-mediated deoxyuridinetriphosphate nick end labeling
2cKO: Double conditional knockout
MDSCs: Myeloid-derived suppressor cells
PD-L1: Programmed cell death ligand 1
PD-1: Programmed cell death protein 1
TME: Tumor microenvironment
ATCC: American Type Culture Collection
CCK8: Cell Counting Kit-8
