# Supplementary material for: Gelatinase-sensitive nanoparticles loaded with photosensitizer and STAT3 inhibitor for cancer photothermal therapy and immunotherapy
Source: J Nanobiotechnology. 2021 Nov 21;19:379. doi: 10.1186/s12951-021-01125-7 (PMC8607679; doi:10.1186/s12951-021-01125-7)
Supplement: Supplementary file 1 — Additional file 1: Table S1. Complete blood panel analysis of mice in the control and ICG, Gel-NPs.Standard deviations are based on 4 mice per group. WBC: white blood cell, RBC: red blood cell, HGB: hemoglobin, HCT: hematocrit, MCV: mean corpuscular volume, MCH: mean corpuscular hemoglobin, MCHC: mean corpuscular hemoglobin concentration, PLT: platelets, RDW: red blood cell distribution width. Figure S1. In vitrocytotoxicity of Gel and ICG against HNSCC cell line CAL27 and normal epithelialcell line HIOEC. (a, c) Cell viability of CAL27 cells with treatment with aseries of concentrations of Gel. (b, d) Cell viability of CAL27 cells withtreatment with a series of concentrations of ICG. Figure S2. Body weight change curves over a span of 30 d after the i.v.injection of PBS, ICG and Gel-NPs. FigureS3. Representative HE stained major organ slice images of mice 30 d after repeating i.v.injection of PBS, ICG and Gel-NPs; scalebars, 50 µm. Figure S4. Expression of Enzyme in Human HNSCC. (a, b) Meta-analysis of recent gene expression profiling for MMP2 and MMP9 by TCGA database (n = 566, HNSCC = 522, Normal = 44). P < 0.001.(c) Over-expression of MMP2 in head and neck squamouscell carcinoma as compared with normal oral mucosa (n = 3); scale bars, 50 µm. Figure S5. Gelatin enzyme expression in Tgfbr1/Pten 2cKO mice. (a) Representative HE staining of Tgfbr1/Pten 2cKO head and neck squamous cell carcinoma tissue (n = 3); scale bar, 50 µm. (b) Immunohistochemically staining indicate increase MMP2 expression in Tgfbr1/Pten 2cKO mice HNSCC (n = 3); scalebars, 50 µm. [file 12951_2021_1125_MOESM1_ESM.docx]

*Additional file 1*

**Gelatinase-sensitive nanoparticles loaded with photosensitizer and STAT3 inhibitor for cancer photothermal therapy and immunotherapy**

Lin-Lin Bu^1,2,#,^*, Han-Qi Wang^1,#^, Yuanwei Pan^3^, Lei Chen^1^, Hao Wu^1^, Xianjia Wu^3^, Chenchen Zhao^3^, Lang Rao^3,^*, Bing Liu^1,2,^*, Zhi-Jun Sun^1,2^^,^*

^1^ The State Key Laboratory Breeding Base of Basic Science of Stomatology (Hubei-MOST) & Key Laboratory of Oral Biomedicine Ministry of Education, School & Hospital of Stomatology, Wuhan University, Wuhan 430079, China.

^2^ Department of Oral and Maxillofacial Head Neck Surgery, School & Hospital of Stomatology, Wuhan University, Wuhan 430079, China.

^3^ Institute of Biomedical Health Technology and Engineering, Shenzhen Bay Laboratory, Shenzhen 518132, China.

^#^ These authors contributed equally to this work.

* Corresponding e-mail: lin-lin.bu@whu.edu.cn (L.-L.B); lrao@szbl.ac.cn (L.R.); liubing9909@whu.edu.cn (B.L.); sunzj@whu.edu.cn (Z.-J.S.)

**Additional Table and Figures**

| Group | Unit | PBS | ICG | Gel-NPs |
| --- | --- | --- | --- | --- |
| WBC | 10^9^/L | 8.36±2.01 | 8.34±2.78 | 7.96±1.51 |
| RBC | 10^12^/L | 12.49±2.11 | 14.32±2.34 | 13.44±2.12 |
| HGB | g/L | 147.89±19.18 | 133.31±17.52 | 140.19±17.26 |
| HCT | CV% | 60.37±4.45 | 55.88±6.44 | 53.32±7.65 |
| MCV | fL | 45.23±3.89 | 50.29±4.13 | 50.99±6.32 |
| MCH | Pg | 19.27±0.88 | 20.12±0.55 | 18.73±0.99 |
| MCHC | g/L | 299.89±61.26 | 279.30±58.88 | 302.13±59.78 |
| PLT | 10^9^/L | 910.63±198.79 | 940.43±210.93 | 887.32±195.20 |
| RDW | CV% | 12.19±0.86 | 11.82±0.80 | 11.39±0.55 |

**Table S1.** Complete blood panel analysis of mice in the control and ICG, Gel-NPs. Standard deviations are based on 4 mice per group. WBC: white blood cell, RBC: red blood cell, HGB: hemoglobin, HCT: hematocrit, MCV: mean corpuscular volume, MCH: mean corpuscular hemoglobin, MCHC: mean corpuscular hemoglobin concentration, PLT: platelets, RDW: red blood cell distribution width.

**
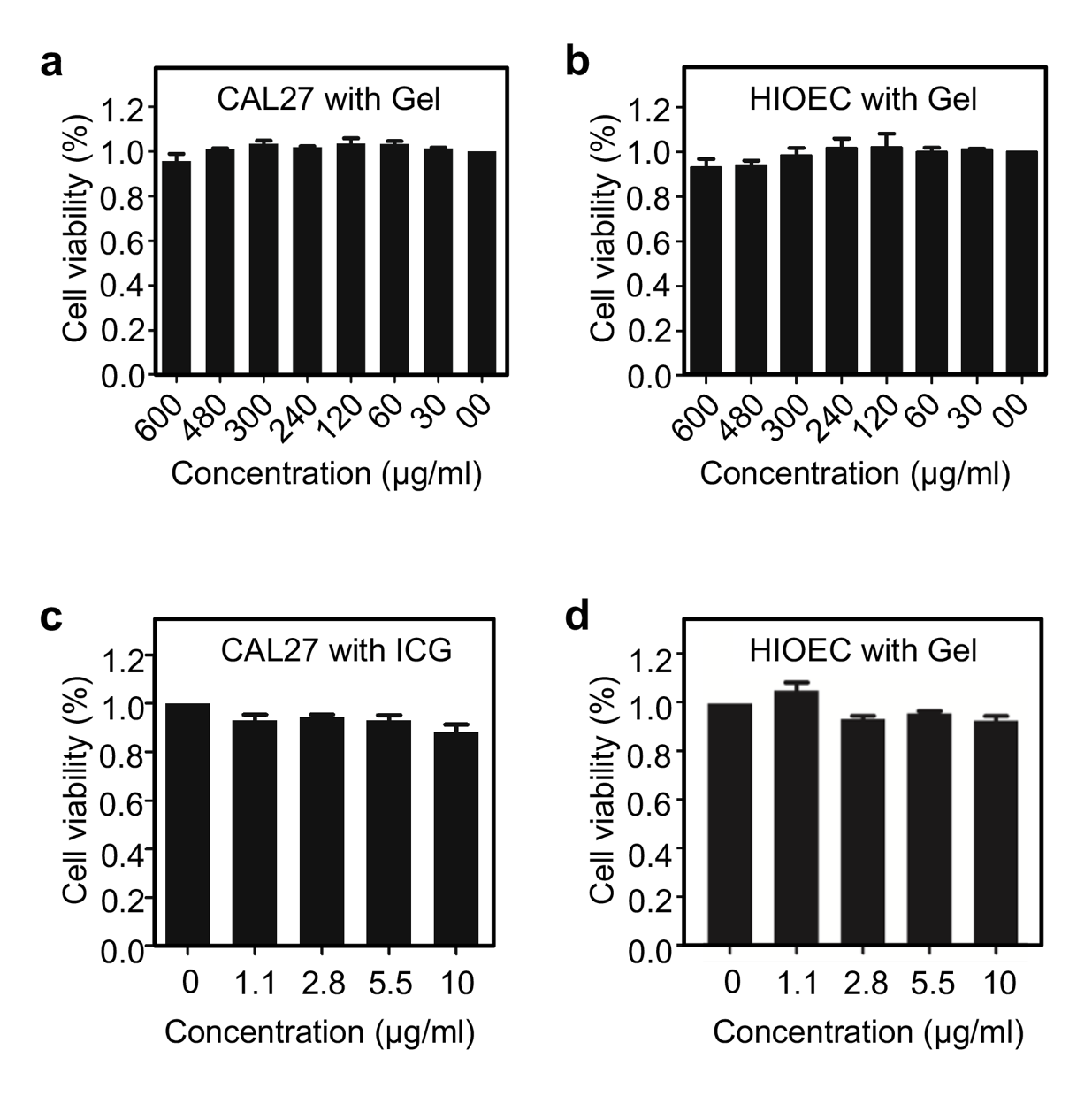
**

**Figure S1.** *In vitro* cytotoxicity of Gel and ICG against HNSCC cell line CAL27 and normal epithelial cell line HIOEC. a, c) cell viability of CAL27 cells with treatment with a series of concentrations of Gel. b, d) cell viability of CAL27 cells with treatment with a series of concentrations of ICG.


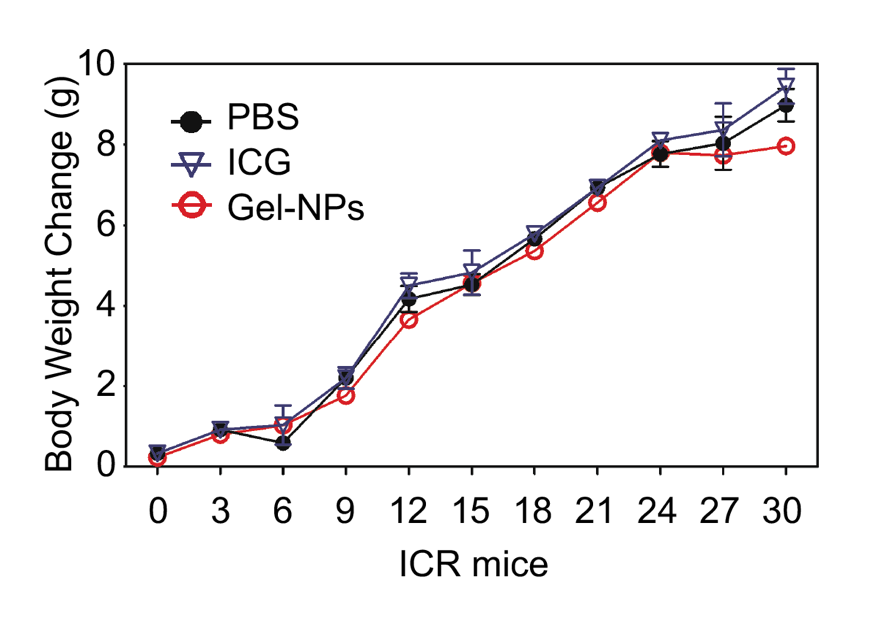


**Figure S2.** Body weight change curves over a span of 30 d after the *i.v.* injection of PBS, ICG and Gel-NPs.


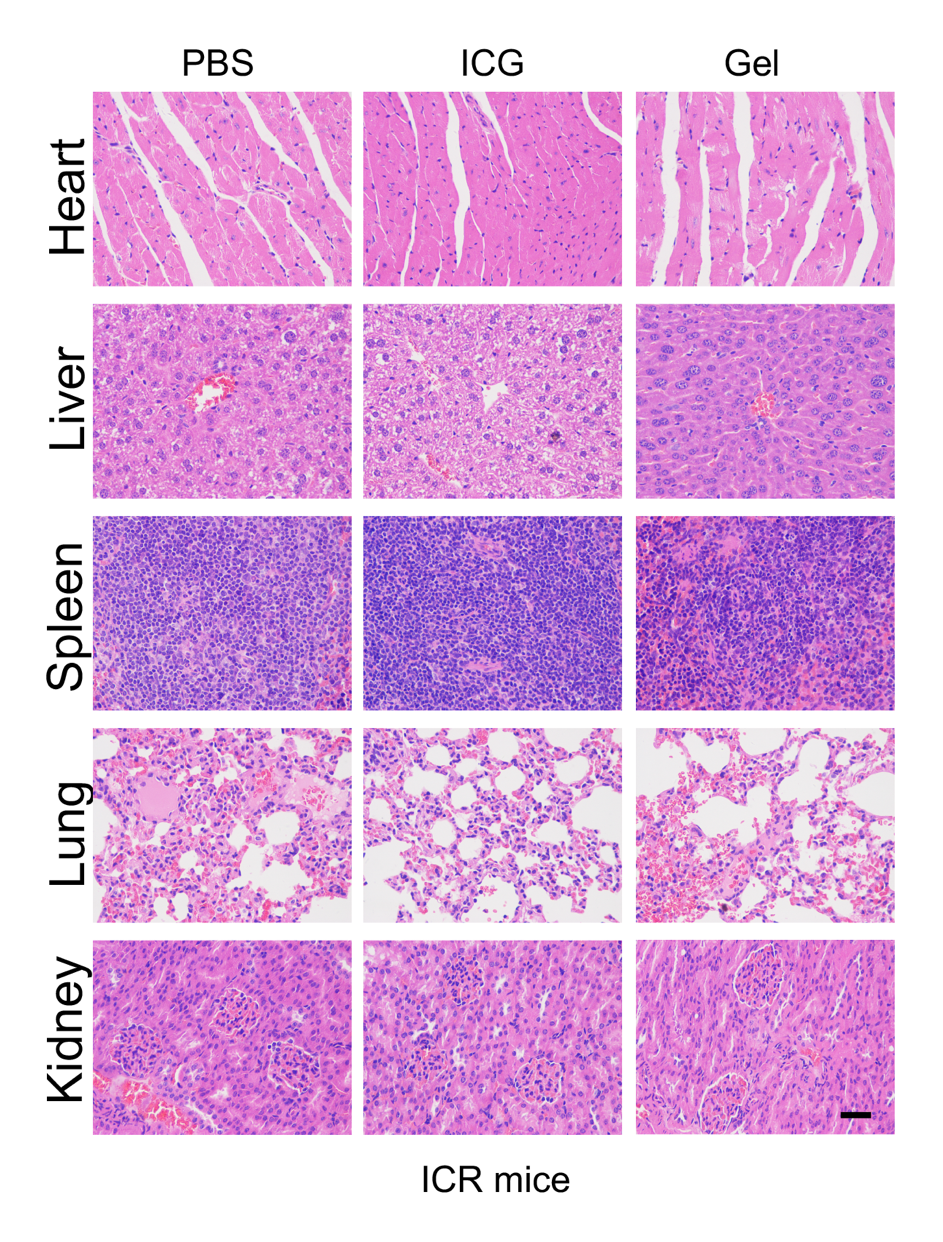


**Figure S3**. Representative HE stained major organ slice images of mice 30 d after repeating *i.v.* injection of PBS, ICG and Gel-NPs; scale bars, 50 µm.


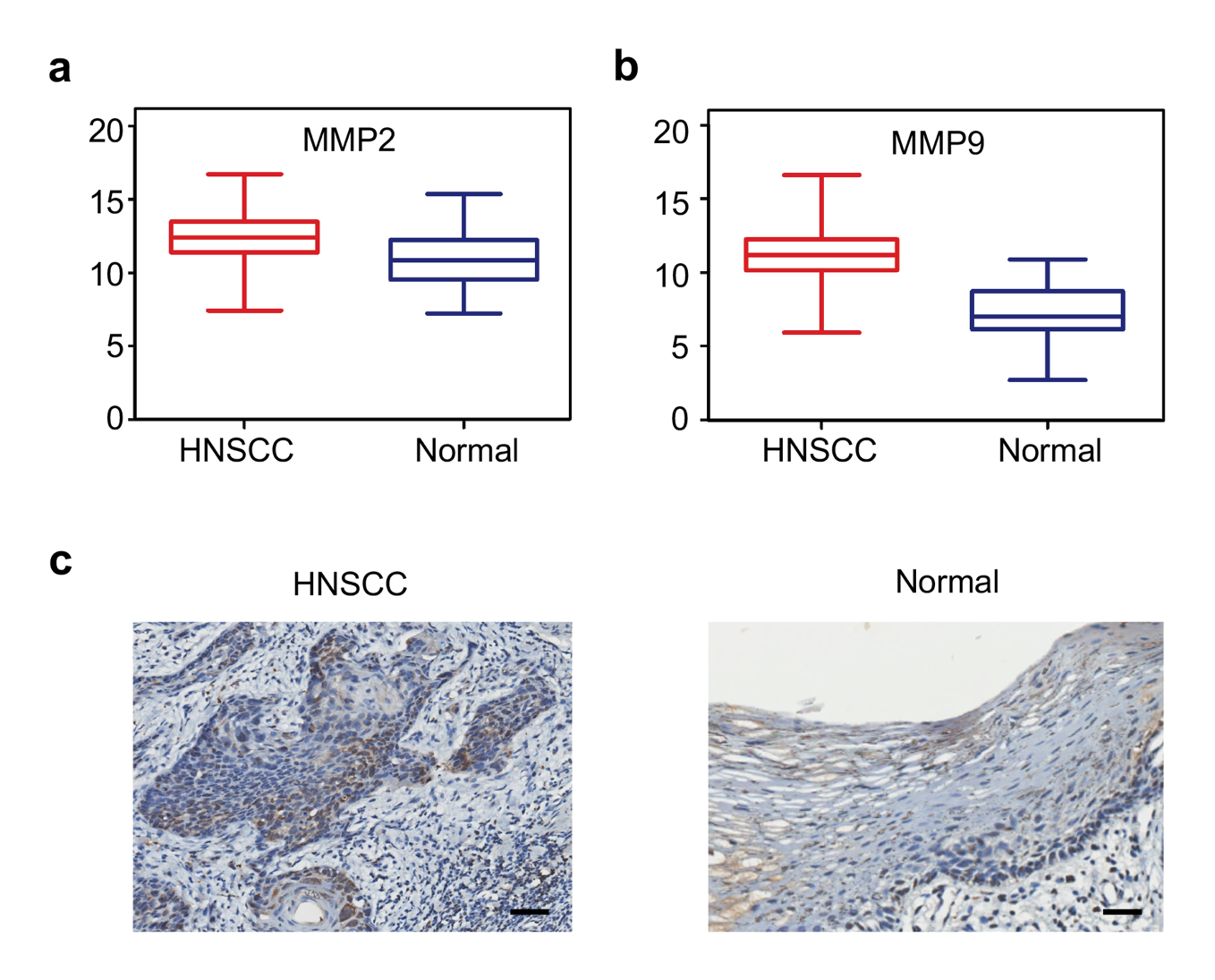


**Figure S4.** Expression of Enzyme in Human HNSCC. a, b) Meta-analysis of recent gene expression profiling for MMP2 and MMP9 by TCGA database (n=566, HNSCC=522, Normal=44). P<0.001. c) Over-expression of MMP2 in head and neck squamous cell carcinoma as compared with normal oral mucosa (n=3); scale bars, 50 µm.


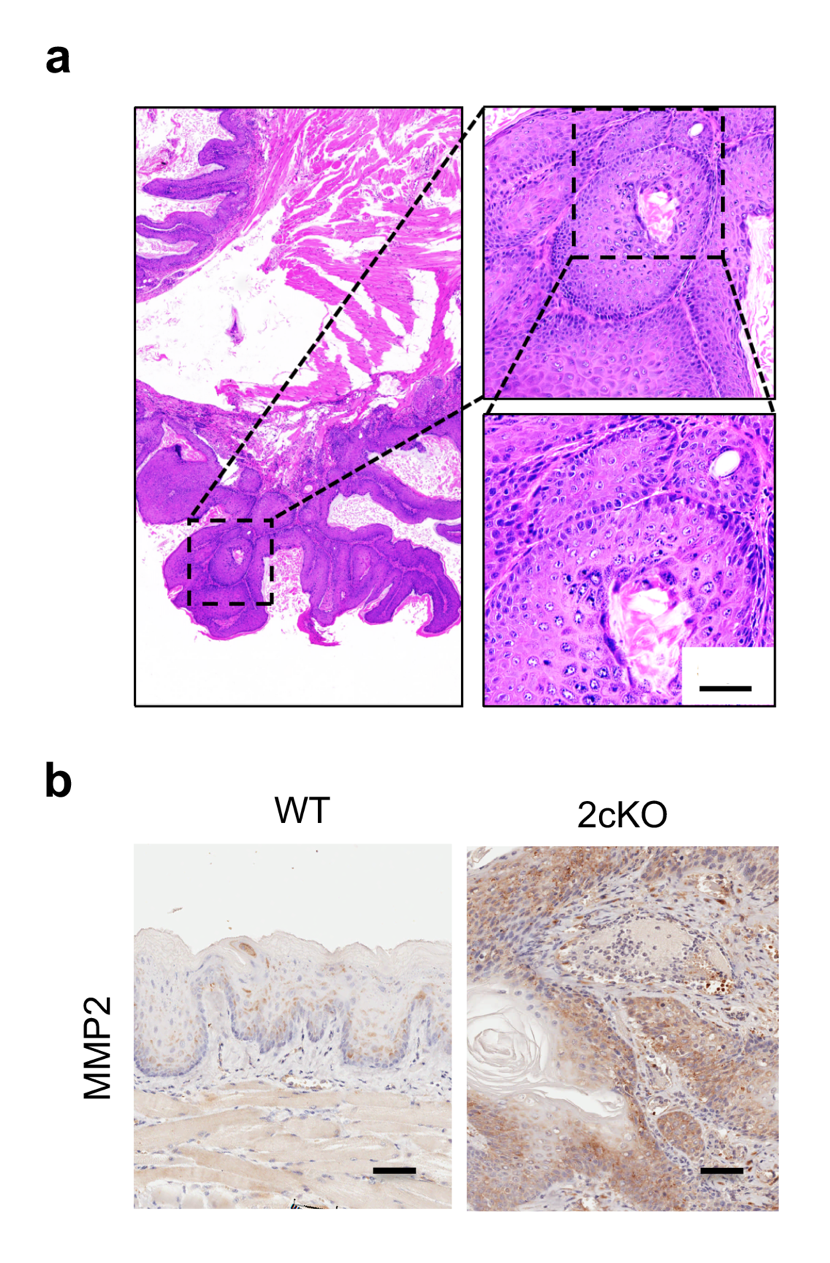


**Figure S5.** **Gelatin enzyme expression in *Tgfbr1/Pten* 2cKO mice.** a) Representative HE staining of *Tgfbr1/Pten* 2cKO head and neck squamous cell carcinoma tissue (n=3); scale bar, 50 µm. b) Immunohistochemically staining indicate increase MMP2 expression in *Tgfbr1/Pten* 2cKO mice HNSCC (n=3); scale bars, 50 µm.
